# From neuromyths to neuroscience literacy: a cross-domain study of brain misconceptions among educators in Chilean special schools

**DOI:** 10.3389/fpsyg.2026.1768430

**Published:** 2026-04-23

**Authors:** Felipe von Hausen, Claudia Carrasco-Manríquez, Denise Quiroz-Martinez, María Josefina Larraín-Valenzuela

**Affiliations:** 1 Facultad de Comunicaciones y Artes, Universidad de Las Américas, Santiago, Chile; 2 Centro de Investigación en Cognición e Inclusión para la Alfabetización Académica en Educación Superior (CIPAES), Universidad de Las Américas, Santiago, Chile; 3 Facultad de Ciencias de la Educación, Universidad de Talca, Linares, Chile; 4 Department of Education, Faculty of Social Sciences, University of Stirling, Stirling, United Kingdom; 5 Centro de Investigación para la Mejora de los Aprendizajes (CIMA), Facultad de Educación, Universidad del Desarrollo, Santiago, Chile

**Keywords:** educational neuroscience, neurodevelopmental misconceptions, neuromyths, science-informed education, teacher beliefs

## Abstract

**Introduction:**

Neuromyths remain widespread among educators, but their distribution may vary across domains and educational contexts. This study investigated brain-related misconceptions among educators working in Chilean special schools, focusing on three complementary domains: general brain knowledge, neurodevelopmental neuromyths, and educational neuromyths.

**Methods:**

We used a quantitative, cross-sectional survey design and collected data from 142 educators through three questionnaires assessing general brain knowledge and misconceptions related to neurodevelopment and education. We first examined prevalence patterns across domains descriptively and then fitted item-level mixed-effects logistic regression models to test whether general brain knowledge, interest in educational neuroscience, and self-perceived neuroscience knowledge were associated with accuracy in identifying misconceptions.

**Results:**

We found a clear cross-domain gradient: educational neuromyths yielded the highest proportion of incorrect responses, neurodevelopmental neuromyths occupied an intermediate position, and general brain knowledge showed the lowest proportion of incorrect responses. At the broad domain level, we found no significant associations between accuracy and general brain knowledge, interest in educational neuroscience, or self-perceived neuroscience knowledge in either neurodevelopmental or educational neuromyths. At the subgroup level, however, we found a more selective pattern: higher general brain knowledge was associated with lower odds of a correct response in attention-deficit/hyperactivity disorder items and with greater odds of a correct response in learning styles items, whereas no significant effects emerged for the remaining subgroups.

**Discussion:**

Neuroscience literacy does not operate as a single, domain-general protective factor against neuromyth endorsement. Instead, its relationship with accuracy appears to depend on the specific type of claim being evaluated. These findings provide updated evidence from Chile and highlight the importance of examining neuromyths in special education settings, where brain- and development-related beliefs may directly shape pedagogical expectations and instructional decisions.

## 1 Introduction

Neuromyths are misconceptions about the brain and learning that circulate widely in educational settings, often arising from oversimplified or distorted interpretations of neuroscientific findings (Organisation for Economic Co-operation and Development, 2002). In practice, these beliefs can influence educational judgments and teaching decisions despite lacking empirical support (Dekker et al., 2012; Howard-Jones, 2014; Newton and Salvi, 2020). Among the most persistent examples are the beliefs that students learn better when instruction aligns with learning styles, that people are mainly “left-brained” or “right-brained,” and that learning is strictly limited by narrow critical periods (Dekker et al., 2012; Kirschner and van Merriënboer, 2013; Pashler et al., 2008). Although these claims have repeatedly been shown to lack empirical evidence, they remain prevalent in teacher education and school practice globally.

Their persistence reflects more than just individual misunderstanding. Teachers often encounter brain-related claims through popular media, commercial programmes, informal professional development, and simplified accounts of scientific findings rather than through ongoing engagement with primary research (Ferrero et al., 2016; Torrijos-Muelas et al., 2021). In this context, ideas that seem intuitive, appealing, or immediately useful for classroom practice can become normalised even when they do not align with current evidence. This helps explain why neuromyths still circulate despite the ongoing development of educational neuroscience as a field (Howard-Jones, 2014; Pasquinelli, 2012).

Research from Latin America indicates that this issue is not solely a European or North American phenomenon. Gleichgerrcht et al. (2015), in a comprehensive regional study, documented significant levels of misconceptions among Latin American teachers and observed variability across countries. Chile stands out as a particularly relevant case in this context. Previous research with Chilean teachers has revealed high levels of neuromyth endorsement and suggests that misconceptions about the brain are already deeply rooted in the local educational environment (Varas-Genestier and Ferreira, 2017). More recently, Armstrong-Gallegos et al. (2023) noted that misconceptions related to neurodevelopmental disorders may be even more widespread than general neuromyths among Chilean educational professionals. Collectively, these findings imply that Chile offers more than a mere replication context: it provides an opportunity to explore how different types of brain-related misconceptions are distributed within a system where such beliefs are already active and pedagogically significant.

This issue becomes particularly important in special education. In Chile, support for students with special educational needs is structured through both special schools and support frameworks within mainstream education, including the *Programa de Integración Escolar* (PIE). Within this system, diagnostic categories, interdisciplinary assessments, curriculum adaptations, and support provision are closely linked to educational decisions. In such settings, teachers work daily with students whose educational paths are often understood through labels such as ADHD, autism spectrum condition, dyslexia, Down syndrome, or general learning disabilities. Consequently, misconceptions about brain development, neuroplasticity, sensory processing, or supposedly optimal teaching methods may directly influence pedagogical expectations, instructional planning, and support choices. For instance, the belief in learning styles may foster rigid assumptions about how specific students should be taught, while deterministic views of developmental timing may reduce expectations about what students can achieve with sustained support (Blakemore and Frith, 2005; Gini et al., 2021; Thomas and Knowland, 2009).

Another reason to focus on special schools is that misconceptions about the brain do not all function in the same way. Some relate to broad foundational knowledge about the brain, while others are more connected to educational practice or developmental and diagnostic explanations. General brain knowledge involves understanding core aspects of brain structure, function, plasticity, and the biological processes involved in learning and memory (Macdonald et al., 2017; Pickering and Howard-Jones, 2007). This knowledge is crucial because teachers are often exposed to claims based on brain science, and critically assessing these claims largely depends on their understanding of basic neuroscience. However, previous research also indicates that accurate knowledge of the brain and the endorsement of neuromyths do not form a straightforward spectrum. Teachers might be familiar with broad neuroscientific concepts while still holding specific misconceptions about instruction or development (Horvath et al., 2018; Macdonald et al., 2017; Papadatou-Pastou et al., 2017).

This distinction is particularly relevant for neurodevelopmental neuromyths. These misconceptions often arise when valid scientific concepts—such as neuroplasticity, sensitive periods, or developmental variability—are transformed into overly deterministic educational claims (Gini et al., 2021; Howard-Jones, 2014; Thomas and Knowland, 2009). In special education, where developmental explanations are especially important, such misconceptions can be particularly influential. At the same time, educational neuromyths like learning styles or hemispheric dominance may be especially resistant because they are ingrained in everyday teaching discourse and frequently presented as practical classroom strategies (Dekker et al., 2012; Newton and Salvi, 2020; Pasquinelli, 2012). For these reasons, considering neuromyth endorsement as a single, undifferentiated construct might obscure important differences between domains.

This study investigates neuromyths across three related areas in educators working in Chilean special schools: general brain knowledge, neurodevelopmental neuromyths, and educational neuromyths. This cross-area approach helps determine whether a common pattern exists across all fields or if some misconceptions are more enduring than others. It also enables us to see whether factors like interest in educational neuroscience and self-assessed neuroscience knowledge consistently relate to accuracy across areas, or if their influence varies depending on the type of claim being assessed.

Our study makes four main contributions. First, it offers updated evidence from Chile, where neuromyth endorsement is well documented but still requires more detailed, domain-specific analysis. Second, it focuses on an important professional group—educators in special schools—whose work is particularly influenced by beliefs related to the brain and development. Third, it compares different areas of neuroscience-related knowledge and misconceptions instead of reducing them to a single score. Fourth, it investigates whether objective and self-reported measures of neuroscience literacy are similarly related to accuracy across various domains. Based on these aims, we address two research questions: (1) how common are neuromyths and gaps in knowledge about the brain among educators in Chilean special schools across different areas, and (2) how are factors such as age, sex, interest in neuroscience, and self-perceived neuroscience knowledge associated with accuracy in identifying these misconceptions? We seek to clarify how neuroscience literacy is organised within a sector of the teaching profession where developmental interpretations of learning are especially relevant and where misconceptions may have direct implications for educational practice.

## 2 Materials and methods

This research adopted a quantitative, cross-sectional survey design to investigate the prevalence of neuromyth beliefs and levels of general brain knowledge among educators working in Special Schools in Chile. This population was selected because teachers in Special Schools frequently rely on developmental and neurocognitive assumptions when planning instruction, and Chilean data indicate a high prevalence of neuromyth beliefs among educators (Varas-Genestier and Ferreira, 2017). We also aimed to explore the relationship between general knowledge of the brain and the endorsement of neuromyths across domains.

We initially recruited a total of 153 educators currently working in Chilean Special Schools. However, after excluding 11 participants who did not consent to participate in the study, the final sample consisted of 142 educators. This approach ensured diverse representation across educational contexts, including early childhood education, basic education, special education, physical education, and educational psychology. Participants’ ages ranged from 23 to 65 years, with a mean age of 39.38 years (SD = 10.29). Age was recorded as a continuous variable and examined descriptively. Its distribution departed from normality (Shapiro–Wilk *W* = 0.954, *p* < 0.001).

The majority of participants were female (83%), reflecting the gender distribution commonly found in the teaching profession [Centro de Estudios MINEDUC (CEM), 2022]. Sex was recorded descriptively; given the marked imbalance in the sample, it was not included in the final mixed-effects models.

We employed three questionnaires in their Spanish versions to assess participants’ beliefs in neuromyths and their general knowledge of the brain. To our knowledge, not all instruments used here have undergone formal psychometric validation in Chilean samples. Accordingly, we treated them as established research questionnaires adapted for use in Spanish-speaking and Chilean educational contexts, and we indicate below the source and adaptation pathway followed for each instrument:

1) Neuromyths in Education Questionnaire: This instrument evaluates common educational neuromyths, such as the belief in learning styles and the misconception that individuals use only 10% of their brain capacity. The questionnaire consists of 26 items, each rated on a 3-point Likert scale (true/false/I do not know), that capture the extent to which participants endorse these widespread misconceptions. In the present study, we used the Spanish version of the educational neuromyth items, following prior Spanish-speaking and Chilean research based on Dekker et al. (2012) (e.g., Varas-Genestier and Ferreira, 2017; Armstrong-Gallegos et al., 2023).
2) Neurodevelopmental Neuromyths Questionnaire (Gini et al., 2021): This questionnaire focuses on neuromyths related to neurodevelopment, including beliefs about critical periods for learning, the effects of early childhood experiences on brain development, and misconceptions about conditions such as ADHD, autism, dyslexia, and Down syndrome. The questionnaire comprises 30 items, rated on a 3-point Likert scale (true/false/I do not know), designed to identify misconceptions that may influence educational and clinical practices. Prior Chilean work using this questionnaire reports that the neurodevelopmental items were translated into Spanish and then back-translated into English to preserve semantic equivalence (Armstrong-Gallegos et al., 2023).
3) General brain knowledge items (Ruhaak and Cook, 2018): General brain knowledge was assessed using the 15 general brain knowledge items reported in Ruhaak and Cook (2018) (see their Table 3), administered with a true/false/I do not know response format. Higher scores indicate a more accurate understanding of basic brain function and neuroscience. In the present study, we used a Spanish version translated by the research team for the Chilean context.

4) The data collection was conducted entirely online to facilitate access and ensure a wide geographical reach across different regions of Chile. Participants were invited to complete the three questionnaires through a secure online platform over a 4-week period to maximise response rates and reduce potential biases associated with time constraints. Each questionnaire was designed to take approximately 15–20 min to complete, with the total participation time not exceeding 1 h. All responses were anonymised, and participants were informed about the confidentiality of their data and the voluntary nature of their participation. Once educators agreed to participate, they completed an ethical consent form, which adhered to the principles outlined in the Declaration of Helsinki (World Medical Association, 2013) for research involving human participants.

### 2.1 Data analysis

We first computed descriptive statistics to examine response distributions, prevalence patterns, and score variation across the three instruments. For scoring purposes, correct responses were coded as 1 and incorrect responses as 0. Total scores were then calculated for each instrument as the sum of correct responses, with higher scores indicating greater accuracy. General brain knowledge was operationalised as the total number of correct responses on the General Knowledge of the Brain questionnaire.

For inferential analyses, we modelled responses at the item level. Each response was coded as correct or incorrect, and I do not know responses were excluded from the mixed-effects analyses so that accuracy could be treated as a binary outcome. We fitted mixed-effects logistic regression models with a binomial family and logit link. General brain knowledge was grand-mean centred before modelling. Self-reported interest in educational neuroscience and self-perceived neuroscience knowledge were collapsed into three levels (high, neutral, low) and entered using sum contrasts, so that the intercept represented the grand mean rather than a single reference category. Objective general brain knowledge and self-perceived neuroscience knowledge were treated as conceptually distinct predictors, because one captures performance-based literacy and the other metacognitive self-assessment.

We fitted separate domain-level models for Neurodevelopmental Neuromyths and Neuromyths in Education. In these models, accuracy was the dependent variable; general brain knowledge, interest, and self-perceived knowledge were entered as fixed effects; and random intercepts for participants and items were included to account for repeated responses and item-level variability. We then fitted additional subgroup models separately for each subgroup within Instrument 1 (ADHD, Autism, Down Syndrome, Dyslexia, and General Learning Disabilities) and Instrument 2 (Brain Function, Cognitive Development, Learning Styles, and Sex Differences). These subgroup models included general brain knowledge as the fixed effect and random intercepts for participants and items. We report results as odds ratios with 95% confidence intervals, using *p* < 0.05 as the criterion for statistical significance. All analyses were conducted in *R*.

## 3 Results

This section presents the study’s findings, including self-reported interest and knowledge in educational neuroscience, the prevalence of neuromyths, and the statistical analysis of predictors of neuromyth endorsement. Descriptive analyses illustrate participants’ engagement with neuroscience, their perceived level of knowledge, and the extent to which neuromyths persist across different domains. Additionally, inferential analyses explore how individual differences in interest and knowledge influence accuracy in identifying neuromyths.

**Figure 1:**
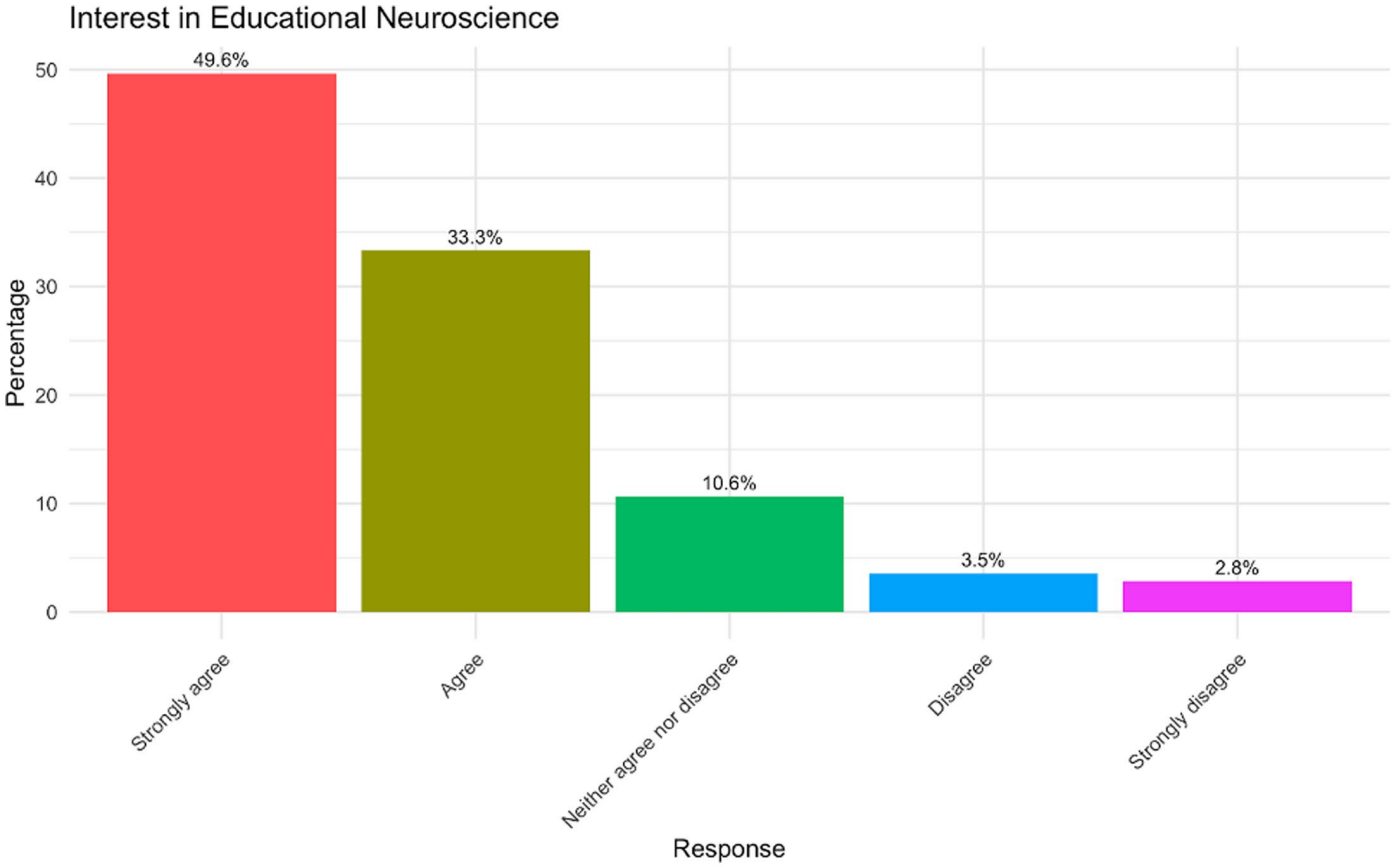
Interest in educational neuroscience.

Figure 1 presents participants’ self-reported interest in educational neuroscience, showcasing a strong inclination towards engagement with this field. Nearly half of the participants (49.6%) indicated that they “Strongly agree” with being interested, making it the most prevalent response category. Additionally, 33.3% selected “Agree,” resulting in a substantial combined majority (82.9%) expressing a positive interest in educational neuroscience. This might suggest a high level of perceived relevance of neuroscience to education among the participants. In contrast, a smaller proportion of respondents were ambivalent, with 10.6% selecting “Neither agree nor disagree”. Disinterest in educational neuroscience was notably low, as evidenced by the minimal percentages selecting “Disagree” (3.5%) and “Strongly disagree” (2.8%).

**Figure 2:**
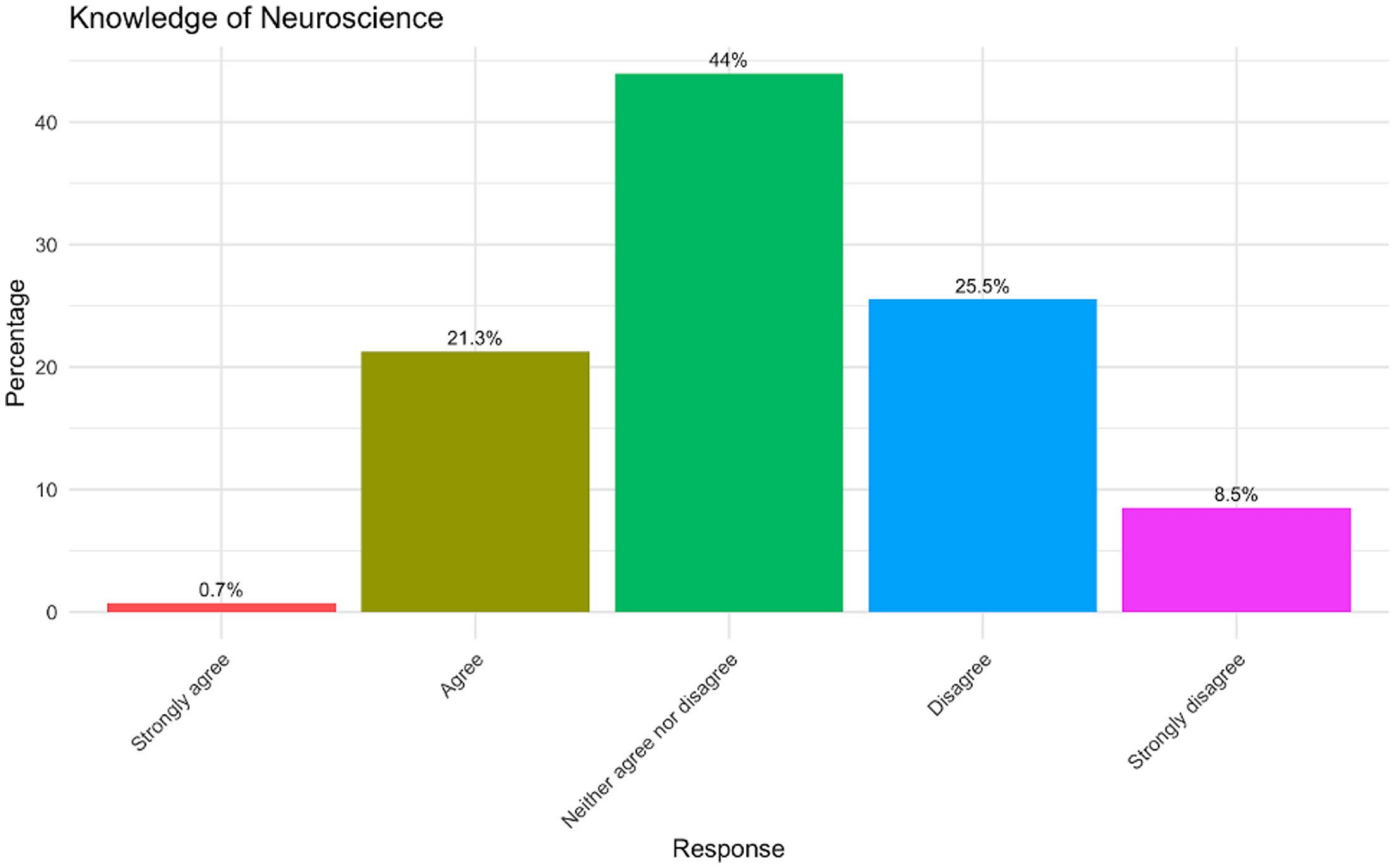
Knowledge of neuroscience.

Figure 2 illustrates participants’ self-reported levels of knowledge in neuroscience with a spectrum of confidence levels across the sample. A notable 44% of participants selected “Neither agree nor disagree,” indicating uncertainty or neutrality regarding their neuroscience knowledge. This is the largest proportion among all response categories, suggesting a significant level of ambivalence in self-perception. Meanwhile, 25.5% disagreed with the statement, and 8.5% strongly disagreed, collectively making up 34% who perceive their neuroscience knowledge as limited. In contrast, 21.3% of participants agreed, reflecting a moderate level of confidence, whereas only 0.7% strongly agreed, indicating a high level of perceived expertise. The low percentage of strong agreement (0.7%) may highlight a potential gap in neuroscience education or exposure within the participant pool. Moreover, the high proportion of neutral responses (44%) may point to participants’ lack of awareness about their own knowledge levels, possibly stemming from limited opportunities to engage with neuroscience concepts.

**Figure 3:**
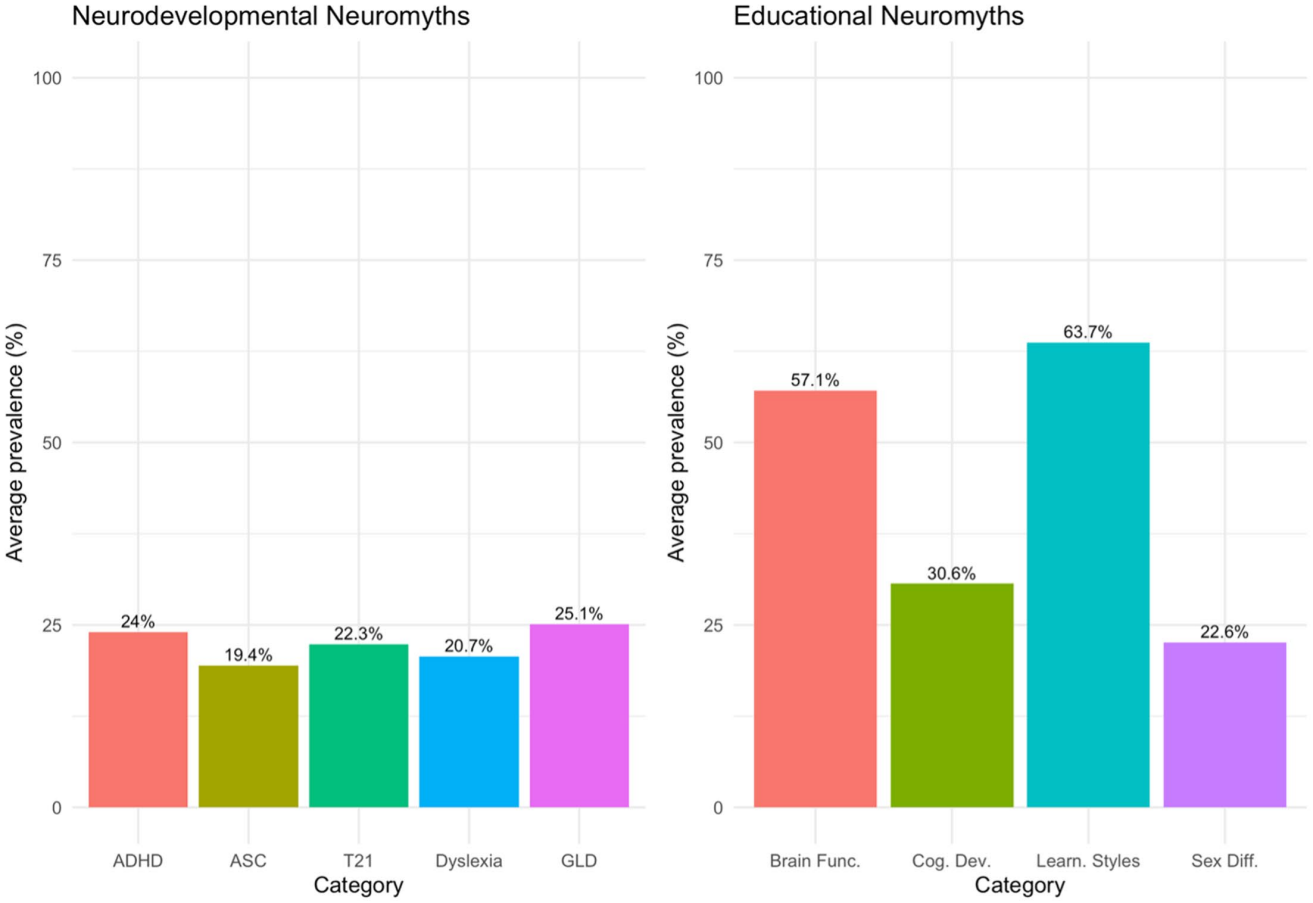
Prevalence of neuromyths.

Figure 3 shows how common neuromyths are in two main areas: neurodevelopmental neuromyths and educational neuromyths. In the neurodevelopmental area, the highest average prevalence was seen with General Learning Disabilities (25.1%), followed by ADHD (24.0%), Down Syndrome (22.3%), Dyslexia (20.7%), and Autism Spectrum Condition (ASC; 19.4%). For ADHD, a common misconception was that reducing dietary sugar helps lessen symptoms, believed by 70.92% of respondents. Another popular misconception was that most children with ADHD will outgrow their symptoms, held by 39.72%. In ASC, overall prevalence was lower, but misconceptions about social rejection and empathy still existed. In Dyslexia, a frequent misconception was that all dyslexic children see letters backwards. In the General Learning Disabilities group, the most widespread misconception was that a multisensory approach is always the best, supported by 77.3% of respondents.

In the educational neuromyth domain, Learning Styles showed the highest average prevalence (63.7%), followed by Brain Function (57.1%), Cognitive Development (30.6%), and Sex Differences (22.6%). The belief that students learn better when instruction matches their preferred sensory modality was among the most highly endorsed misconceptions, as was the idea that visual learners benefit most from audiovisual materials. In the Brain Function category, the outdated notion of left-brain versus right-brain dominance remained especially common. Cognitive Development myths showed a lower overall prevalence, although some misconceptions about critical periods and enriched environments persisted. Sex Differences myths were the least prevalent among the educational categories, though some gender-based assumptions about verbal, visual, and kinesthetic learning still existed.

**Figure 4:**
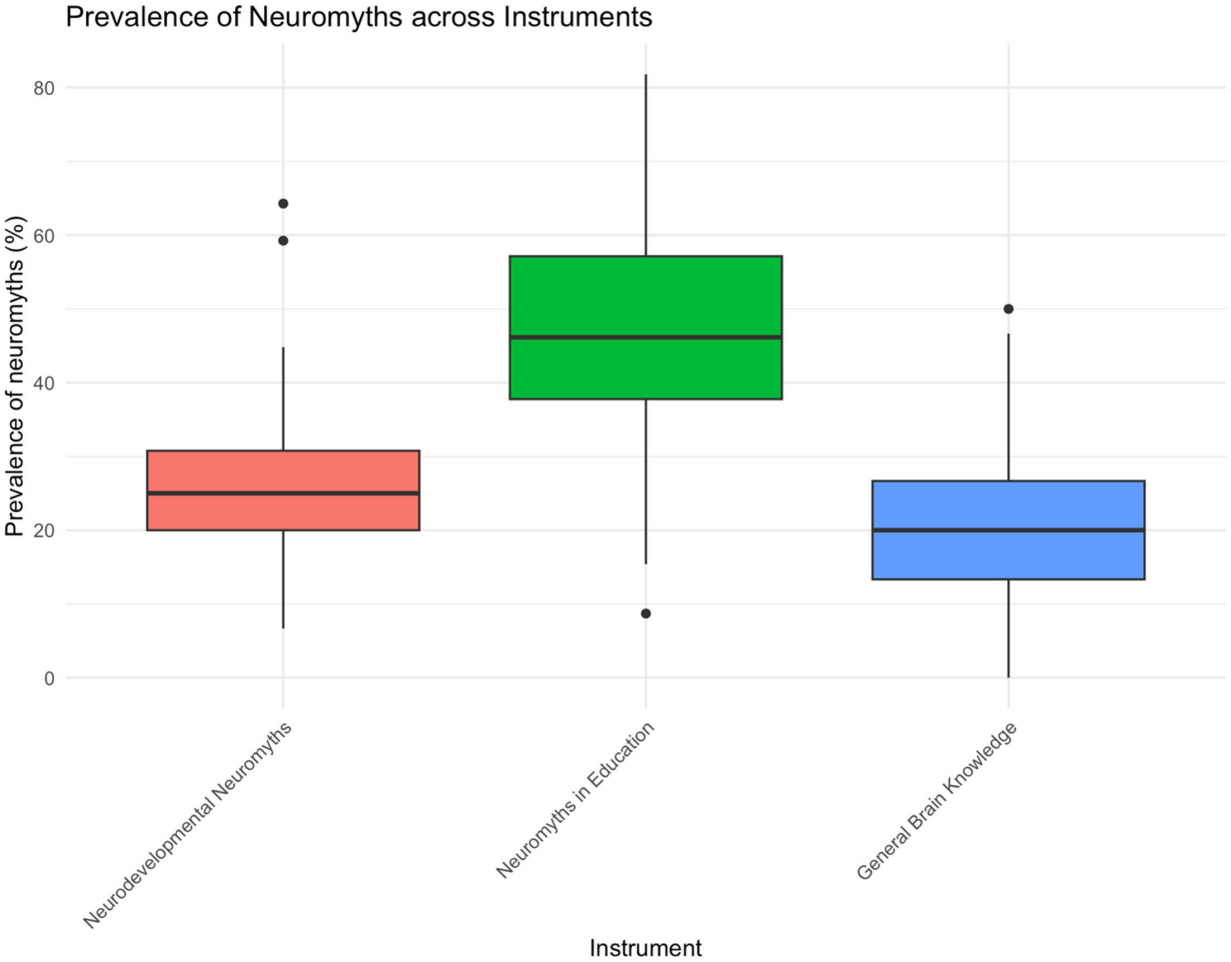
Prevalence of neuromyths across instruments.

Figure 4 shows the distribution of incorrect responses across the three domains assessed in the study: Neurodevelopmental Neuromyths, Neuromyths in Education, and General Brain Knowledge. General Brain Knowledge showed the lowest proportion of incorrect responses overall (median = 20.0%, mean = 19.6%, SD = 11.5), indicating comparatively better performance in this domain. Neurodevelopmental Neuromyths showed an intermediate level of incorrect responses (median = 25.0%, mean = 26.3%, SD = 9.25), whereas Neuromyths in Education showed the highest level (median = 46.2%, mean = 46.7%, SD = 14.2). This pattern indicates that participants were relatively more accurate when responding to general statements about brain function than when evaluating claims related to education, with neurodevelopmental misconceptions occupying an intermediate position. A Friedman test confirmed significant differences across the three domains, *χ*^2^(2) = 168.01, *p* < 0.001. Pairwise Wilcoxon signed-rank tests with Holm correction showed that all domain comparisons were significant: Neurodevelopmental Neuromyths vs. Neuromyths in Education, p < 0.001; Neurodevelopmental Neuromyths vs. General Brain Knowledge, *p* < 0.001; and Neuromyths in Education vs. General Brain Knowledge, *p* < 0.001. Overall, the results indicate a clear gradient across domains, with the greatest concentration of incorrect responses in educational neuromyths and the lowest in general brain knowledge.

### 3.1 Statistical analysis

For inferential analyses, responses were modelled at the item level. Each response was coded as correct or incorrect, and I do not know responses were excluded from the mixed-effects models so that accuracy could be treated as a binary outcome. General brain knowledge was operationalised as the total number of correct responses on the General Brain Knowledge instrument. This score was grand-mean centred before modelling. Self-reported interest in educational neuroscience and self-perceived neuroscience knowledge were collapsed into three levels (high, neutral, low) and entered using sum contrasts, so that the intercept represented the grand mean rather than a single reference category.

Mixed-effects logistic regression models with a binomial distribution and logit link were fitted separately for the two main domains: Neurodevelopmental Neuromyths and Neuromyths in Education. These models included general brain knowledge, interest, and self-perceived knowledge as fixed effects, and random intercepts for participants and items to account for repeated responses and item-level variability. To explore whether the effect of general brain knowledge differed across specific types of misconceptions, additional mixed-effects logistic regression models were fitted separately for each subgroup within Instrument 1 (ADHD, Autism, Down Syndrome, Dyslexia, and General Learning Disabilities) and Instrument 2 (Brain Function, Cognitive Development, Learning Styles, and Sex Differences). These subgroup models included general brain knowledge as the fixed effect and random intercepts for participants and items. Results are reported as odds ratios (ORs) with 95% confidence intervals (CIs), with statistical significance set at *p* < 0.05.

At the domain level, the mixed-effects models showed that general brain knowledge, interest in educational neuroscience, and self-perceived neuroscience knowledge were not significantly associated with accuracy in either broad domain. In the neurodevelopmental domain, general brain knowledge was not a significant predictor of accuracy (OR = 0.997, 95% CI [0.963, 1.033], *p* = 0.870). Likewise, neither interest nor self-perceived knowledge showed significant deviations from the grand mean. A similar pattern was observed in the educational domain, where general brain knowledge was also non-significant (OR = 1.003, 95% CI [0.950, 1.059], *p* = 0.902), and no significant effects emerged for interest or self-perceived knowledge.

At the subgroup level, as shown in Table 1, however, the effect of general brain knowledge was more selective. Within Neurodevelopmental Neuromyths, a significant association was observed only for ADHD items, where higher general brain knowledge was associated with lower odds of a correct response (OR = 0.898, 95% CI [0.839, 0.960], *p* = 0.002). No significant effects were found for Autism, Down Syndrome, Dyslexia, or General Learning Disabilities. Within Neuromyths in Education, a significant positive association emerged only for Learning Styles items, such that higher general brain knowledge was associated with greater odds of correctly identifying these misconceptions (OR = 1.193, 95% CI [1.046, 1.360], *p* = 0.008). No significant effects were observed for Brain Function, Cognitive Development, or Sex Differences. Taken together, these findings indicate that general brain knowledge did not operate as a broad, domain-general predictor of performance. Instead, its association with accuracy was specific to particular types of misconceptions, with the clearest positive pattern observed for Learning Styles items.

**Table 1:** Mixed-effects logistic regression models for subgroup analyses.

Domain	Subgroup	*b*	SE	*z*	*p*	OR	95% CI for OR
Neurodevelopmental neuromyths	ADHD	−0.108**	0.034	−3.134	0.002	0.898	[0.839, 0.960]
	Autism	−0.016	0.051	−0.310	0.757	0.984	[0.890, 1.089]
	Down syndrome	0.054	0.049	1.107	0.268	1.056	[0.959, 1.162]
	Dyslexia	0.050	0.050	1.006	0.314	1.051	[0.954, 1.159]
	General learning disabilities	0.034	0.025	1.350	0.177	1.035	[0.985, 1.087]
Educational neuromyths	Brain function	−0.087	0.114	−0.764	0.445	0.917	[0.733, 1.146]
	Cognitive development	−0.054	0.042	−1.273	0.203	0.948	[0.873, 1.029]
	Learning styles	0.176**	0.067	2.639	0.008	1.193	[1.046, 1.360]
	Sex differences	−0.004	0.043	−0.089	0.929	0.996	[0.916, 1.083]

Coefficients represent the effect of centered general brain knowledge score. All models included random intercepts for participants and items. OR, odds ratio; CI, confidence interval. *p < 0.05, **p < 0.01, ***p < 0.001.

## 4 Discussion

This study investigated neuromyth endorsement among teachers in Chilean special schools across three domains: general brain knowledge, neurodevelopmental neuromyths, and educational neuromyths. The evidence shows a distinct cross-domain gradient, with educational neuromyths yielding the highest proportion of incorrect responses, neurodevelopmental neuromyths occupying an intermediate position, and general brain knowledge yielding the fewest errors. This pattern underscores that neuroscience literacy is not a singular construct. Educators may demonstrate strong core knowledge while simultaneously endorsing specific misconceptions prevalent in pedagogical discourse. These results correspond with previous research, indicating that accurate neuroscience knowledge and neuromyth endorsement can coexist rather than being mutually exclusive (Horvath et al., 2018; Macdonald et al., 2017; Papadatou-Pastou et al., 2017).

At the broad domain level, mixed-effects models did not reveal significant associations between aggregate accuracy and general brain knowledge, interest in educational neuroscience, or self-perceived neuroscience knowledge. This finding indicates that neither objective knowledge nor subjective confidence provides general protection against neuromyth endorsement. Conceptual implications arise from this result, inasmuch as it challenges a simple deficit model by demonstrating that increased knowledge does not necessarily reduce misconceptions. Instead, the evidence supports a more complex perspective: susceptibility to neuromyths depends on the specific claim and its prominence within professional discourse. This interpretation is consistent with prior studies showing that teachers may express strong interest in neuroscience while maintaining inaccurate beliefs about learning and the brain (Ferrero et al., 2016; Torrijos-Muelas et al., 2021).

Educational neuromyths demonstrated particular resistance, representing one of the study’s most significant findings. This outcome is consistent with the international literature, which indicates that myths such as learning styles and hemispheric dominance remain widespread among teachers throughout various countries and educational levels (Dekker et al., 2012; Gleichgerrcht et al., 2015; Newton and Salvi, 2020). Notably, the learning styles myth warrants special attention. Newton and Salvi (2020) found that belief in modifying instruction to students’ preferred learning styles is especially pervasive among educators. In the present data, this domain was also prominent descriptively and, crucially, yielded the clearest positive inferential result: higher general brain knowledge correlated with more accurate responses in the Learning Styles subgroup. This finding indicates that although broad neuroscience knowledge may not uniformly counter all misconceptions, it can facilitate the rejection of particularly prominent and ostensibly ‘brain-based’ pedagogical myths.

This finding possesses particular significance within special education. In mainstream teaching contexts, the learning-styles belief is problematic because it encourages instructors to customise instruction to presumed sensory preferences, despite a lack of empirical support (Pashler et al., 2008; Kirschner and van Merriënboer, 2013). In special schools, the consequences may be even more pronounced. Given that instruction is already structured around student differences, support needs, and developmental profiles, the learning-styles myth is able to reinforce inflexible assumptions regarding appropriate instructional techniques for specific students. Rather than broadening educational opportunities, it may constrain them by promoting fixed sensory classifications and limiting instructional approaches. Thus, this study offers a context-specific contribution: in Chilean special schools, educational neuromyths are not only prevalent but may also have particularly direct pedagogical consequences.

The neurodevelopmental domain exhibited a distinct profile. It was less prone to errors than educational neuromyths but more vulnerable than general brain knowledge. This pattern confirms the view that neurodevelopmental neuromyths frequently stem from reducing valid scientific concepts, such as plasticity, sensitive periods, and developmental variability, into deterministic educational claims (Blakemore and Frith, 2005; Thomas and Knowland, 2009; Gini et al., 2021). This issue is especially relevant in Chilean special education, where teachers routinely work with students whose educational trajectories are closely linked to diagnostic categories and developmental interpretations. In this context, misconceptions regarding ADHD, autism, dyslexia, Down syndrome, or learning disabilities may become ingrained in everyday pedagogical decision-making. The findings thus support the approach of analyzing neurodevelopmental neuromyths as a separate domain rather than aggregating them into a single neuromyth score. This approach is also consistent with Armstrong-Gallegos et al. (2023), who emphasized the need for specific attention to neuromyths related to neurodevelopmental disorders in Chile.

The negative association identified in the ADHD subgroup—where higher general brain knowledge correlated with lower odds of a correct response—presents interpretive challenges. This result warrants cautious interpretation. It does not imply that educators with greater knowledge of the brain have less understanding of ADHD. A more plausible explanation is that ADHD-related items may activate entrenched beliefs prevalent in public and professional discourse, which are not effectively countered by general neuroscience knowledge. ADHD is highly visible in educational discussions, and beliefs regarding diet, behaviour, symptoms, and developmental trajectories often have strong intuitive appeal. In such cases, broad brain knowledge may be insufficient to counteract condition-specific misconceptions. Therefore, this finding is best viewed as evidence of the limitations of domain-general knowledge, rather than as a simple inverse relationship between knowledge and understanding.

The remaining subgroup models yielded non-significant results, which are nonetheless informative. These findings reinforce the conclusion that general knowledge of the brain does not serve as a broad, domain-general predictor of myth rejection. Instead, its impact is selective and dependent on the specific claim. This stresses the value of the study’s cross-domain design. If the analysis had relied solely on a single total score, the conclusion might have been that neuroscience knowledge is inconsequential. By distinguishing between domains and subgroups, the study reveals a more nuanced pattern: general brain knowledge offers limited explanatory power overall, but meaningful associations arise for particular misconceptions. This evidence supports the view that neuroscience literacy among teachers should be conceptualised as multidimensional rather than monolithic (Horvath et al., 2018; Macdonald et al., 2017).

These findings have significant implications for educational practice, particularly in the Chilean and broader regional contexts described in the introduction. Given the persistence of neuromyths among Chilean teachers, as observed beyond Europe and North America (Gleichgerrcht et al., 2015; Varas-Genestier and Ferreira, 2017), the study underscores the need for targeted teacher education. Educational programs and in-service training should prioritize addressing persistent misconceptions, such as learning styles and neurodevelopment-related beliefs, rather than focusing exclusively on general neuroscience content. This targeted strategy is more likely to mitigate the practical impact of neuromyths in special education classrooms. These implications highlight the importance of designing interventions that directly confront the most consequential misconceptions in context, as supported by recent research demonstrating the effectiveness of focused myth correction (Ferrero et al., 2020; Ruiz-Martin et al., 2022).

Several limitations should be acknowledged. The cross-sectional design precludes causal inference, and questionnaire data cannot ascertain the extent to which these beliefs influence actual classroom practice. Additionally, self-reported interest and self-perceived knowledge are subjective and should not be conflated with formal training or demonstrated expertise. Some subgroup analyses are based on smaller effective sample sizes and should be interpreted with caution. Nevertheless, the study possesses notable strengths: it targets a professionally relevant population, differentiates among various domains of misconception, and models responses at the item level while accounting for clustering by participants and items. These methodological features enable a more precise understanding of how neuroscience-related misconceptions are structured within a Chilean special education context.

This study provides updated evidence that neuromyths remain highly prevalent among educators in Chilean special schools and demonstrates that these misconceptions are not uniformly distributed. Educational neuromyths were the most persistent, neurodevelopmental neuromyths exhibited an intermediate pattern, and general brain knowledge was associated with the fewest errors. Inferential analyses revealed that general knowledge of the brain, interest in educational neuroscience, and self-perceived neuroscience knowledge did not predict overall accuracy across domains. Instead, general brain knowledge had a selective role: it was positively associated with correct responses in the Learning Styles subgroup and negatively associated with correct responses in the ADHD subgroup, with no significant effects observed in the other subgroups.

Overall, the present findings support a multidimensional view of neuroscience literacy in education. Teachers may show relatively accurate general knowledge about the brain while still endorsing misconceptions that are pedagogically appealing, culturally entrenched, or closely linked to professional discourse. In the context of Chilean special schools, this pattern is particularly relevant because such beliefs may shape expectations, instructional decisions, and support practices for students whose educational trajectories are already interpreted through developmental and diagnostic frameworks. Rather than treating neuromyth endorsement as a single, undifferentiated phenomenon, our results suggest the need to address those misconceptions that are most persistent and most likely to influence practice in context.

## Data Availability

The datasets presented in this study can be found in online repositories. This data can be found here: https://osf.io/dbf7r/overview?view_only=8aef6a54327c479493d3a5f519703c13.
